# Characteristics and Photovoltaic Applications of Au-Doped ZnO–Sm Nanoparticle Films

**DOI:** 10.3390/nano11030702

**Published:** 2021-03-11

**Authors:** Muhammad Saleem, Kashif Irshad, Saif Ur Rehman, M. Sufyan Javed, Mohd Abul Hasan, Hafiz Muhammad Ali, Amjad Ali, Muhammad Zeeshan Malik, Saiful Islam

**Affiliations:** 1 Institute of Physics, The Islamia University of Bahawalpur, Bahawalpur 63100, Pakistan; 2 Center of Research Excellence in Renewable Energy, King Fahd University of Petroleum & Minerals, Dhahran 31261, Saudi Arabia; kashif.irshad@kfupm.edu.sa (K.I.); amjad.ali@kfupm.edu.sa (A.A.); 3 Department of Physics, COMSATS University Islamabad Lahore Campus, Lahore 54000, Pakistan; saifuetian@gmail.com; 4 School of Physical Science and Technology, Lanzhou University, Lanzhou 730000, China; 5 Civil Engineering Department, College of Engineering, King Khalid University, Abha 61421, Saudi Arabia; mohad@kku.edu.sa (M.A.H.); sfakrul@kku.edu.sa (S.I.); 6 Mechanical Engineering Department, King Fahd University of Petroleum and Minerals, Dharan 31261, Saudi Arabia; hafiz.ali@kfupm.edu.sa; 7 Faculty of Automation, Huaiyin Institute of Technology, Huai’an 223003, China; malik4one@yahoo.com

**Keywords:** Au-doped ZnO–Sm nanoparticles, optical properties, dye-sensitized solar cells, I–V measurements

## Abstract

Au-doped ZnO–samarium nitrate (Sm) nanoparticles with fixed concentrations of Sm (1 wt %) and various concentrations of Au (0.0, 0.5, 1.0 and 1.5 wt %) were prepared and used as photoelectrodes to enhance the photovoltaic efficiency of dye-sensitized solar cells (DSSCs). The cell fabricated with 1.5 wt % of Au-doped ZnO–Sm nanoparticles film achieved an optimal efficiency of 4.35%, which is about 76% higher than that of 0.0 wt % of Au-doped ZnO–Sm-based cell (2.47%). This increase might be due to the formation of a blocking layer at the ZnO–Sm/Au interface, which inhibits the recombination of electrons. This increase may also be attributed to the addition of rare-earth ions in ZnO to enhance the non-absorbable wavelength region of light via up/down-conversion of near-infrared and ultraviolet radiations to visible emission and reduce the recombination loss of electron in the cell. The efficiency of cells may be increased by the blocking layer and up/down-conversion process and thus promote the overall performance of the cells. This work indicates that Au-doped ZnO–Sm nanoparticle films have the potential to increase the performance of DSSCs.

## 1. Introduction

Nanoparticles are relatively vital due to their higher optical, physical and chemical properties. Their optical properties are narrated to be suspended on the size, which transmits exclusive shade due to absorption in visible vicinity [1]. Furthermore, the fluorescence, dimension tune capacity and excessive extinction coefficient make them fairly traumatic in many fields, such as digital devices, nanomedicine and dye-sensitized solar cells [2]. Dye-sensitized solar cells (DSSCs) are fabricated by nanoparticles of ZnO, TiO_2_, CdSe and PbSe as electrode materials because of a large internal surface area for dye adsorption [3,4]. Among these, ZnO is prominent due to its large bandgap (3.37 eV) and large free excitation-binding energy (60 meV) [5]. Currently, researchers are fabricating ZnO-based DSSCs by manipulating the constructions of photoanodes to allow quick electron transport, tremendous light-harvesting, and excessive dye-loading [6,7,8,9]. ZnO is considered a well-known photovoltaic material; however, there are few challenges to upgrade its photovoltaic properties [10]. First, the recombination of photo-generated electrons seems to occur more frequently in ZnO electrodes, which deteriorates the cell performance [11,12,13]. Second, conventionally N719 dye-loaded photoanode of ZnO is functional under visible light. It is well-known that ruthenium dyes, such as N719, N749 have a bandgap of 1.8 eV and can absorb only a visible portion of light. Nevertheless, the greater part of the electromagnetic spectrum consists of ultraviolet (UV) and near-infrared (NIR) light [14]. It means 50% of solar irradiation in the UV and NIR regions is not utilized by ZnO-based DSSCs. An effective way to control these drawbacks of ZnO is doping with other materials. Doping of splendid elements in ZnO is a tremendous way to alter their electrical and optical properties [15]. In current years, rare-earth (RE)-doped nanoparticles have attracted researchers because of their intra 4f transition optical characteristics [16]. Doping of RE ions into ZnO lattice solves the problem of the non-absorbable wavelength region of light by DSSC through the up/down-conversion process. Moreover, the conversion of NIR and UV radiation to visible emission also increases electron transport [17,18,19,20,21,22]. The doping of Au ions modifies the optical absorption and makes a blocking layer for the electron, and reduces the recombination rate [23,24,25,26].

In the recent work, the effect of samarium nitrate (Sm) and Au doping on ZnO is studied with a fixed concentration of Sm (1 wt *%*) and different concentrations of Au (0.0, 0.5, 1.0 and 1.5 wt *%*). ZnO is used as a host material, Au and Sm are the dopant materials. The optical, structural, and photovoltaic characteristics of Au-doped ZnO–Sm are discussed the very first time to increase the efficiency of DSSCs. In this regard, many publications have been published [27,28,29,30] showing doping of Au/Sm/Eu/Ce in ZnO/TiO_2_ separately. The doping of these materials indicates the fast transfer of charge, increased charge separation and utilization of non-absorbable wavelength regions of UV and NIR radiations through the up/down-conversion process. However, none of these reports explain the combined effect of Au and Sm doping in ZnO (Au-doped ZnO–Sm)-based DSSCs. It is seen that the doping of Au and Sm solves the aforementioned problems of ZnO-based DSSCs and improves the overall performance of the device.

## 2. Experimental Details

### 2.1. Preparation of Au-Doped ZnO–Sm Nanoparticles

**Figure 1:**
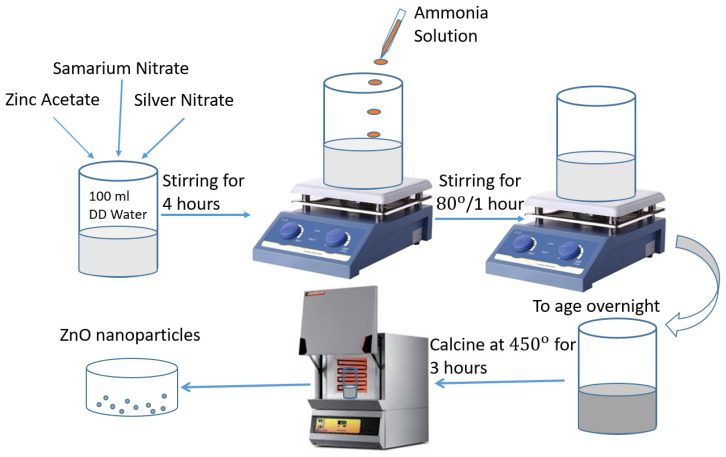
Schematic preparation of Au (0.0, 0.5, 1.0 and 1.5 wt %)-doped ZnO–Sm nanoparticles.

All the regents, zinc acetate, samarium nitrate and silver nitrate, were taken as starting materials and were purchased from Sigma-Aldrich (purity 99.5%) and used as it received. The synthesis detailed for the preparation of Au-doped ZnO–Sm was as follows: 100 wt *%* of zinc acetate, 1 wt *%* of samarium nitrate (Sm (NO_3_)_3_ and (0.0, 0.5, 1.0 and 1.5 wt *%*) of silver nitrate Au (NO_3_)_3_ as given in Table 1. These materials were dissolved in deionized water in a 500 mL beaker under vigorous stirring for 4 h. Then, ammonia (NH_3_) solution was mixed drop-by-dop under constant stirring until the pH of the solution became 8 and continued stirring at 80 °C for another 1 h. After being stirred for 1 h, the well-mixed solution was converted into a thick gel form, and the obtained gel was set aside overnight to age. The gel was dried at 150 °C for 12 h and finally calcine at 450 °C for 3 h. Following the procedure shown in Figure 1, the experiment was repeated three times by changing the concentration of Au.

### 2.2. Characterization

The structural analysis of the prepared nanoparticles was checked using X-ray diffraction of Philips (PAN-alytical X’Pert PRO MRD PW3040, Oberkochen, Germany) diffraction meter from 20–80 degrees. The morphology of the as-grown nanoparticles was imaged with a field emission scanning electron microscope (FE-SEM, JEOL, JSM-6700F, München, Germany). UV-vis spectrophotometer (Lambda-750, Perkin Elmer, Waltham, MA, USA) was employed for the measurement of both absorption and transmittance spectra of the nanoparticles. Photocurrent–voltage (J–V) characteristic of the assembled DSSCs was measured by a Keithley 2400 source meter (Photo Emission Tech Inc., Moorpark, CA, USA) under 1 sun illumination (AM 1.5 G), and the output power was 100 mW/cm^2^, with a PC controlled system. Electrochemical impedance spectroscopy (EIS) was done by an AutoLab electrochemical workstation (Metrohm Autolab PGSTAT302N, Utrecht, The Netherlands) at the open-circuit condition in the dark. The magnitude of the alternate signal was 10 mV.

### 2.3. Preparation of Photo Anode and DSSC Fabrication

**Figure 2:**
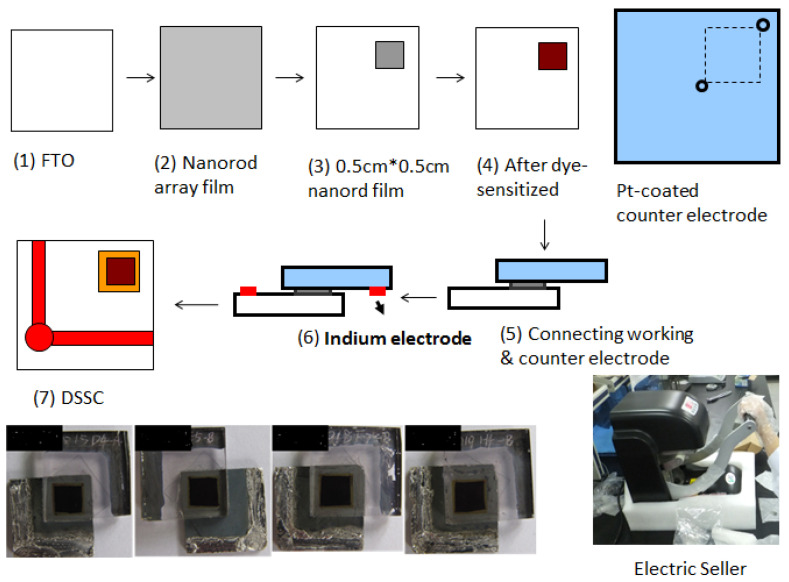
Schematic illustration of Au (0.0, 0.5, 1.0 and 1.5 wt %)-doped ZnO–Sm solar cells.

Prior to the fabrication of Au-doped ZnO–Sm films, FTO glass (3 cm×3 cm) pieces were washed by sonication in detergent, acetone and deionized water each for 12 min and dried in hot air with the help of a hairdryer. The colloidal paste was used for the preparation of working electrodes by using the doctor blade method. The paste was prepared by grinding 4 g of the as-prepared powder in 0.8 mL ethanol using a mortar and pestle. Ethanol was added dropwise in the powder, and surfactant triton X100 was mixed to facilitate the spreading of paste on FTO substrates. The paste area of the working electrode was 1.6 cm × 1.6 cm = 2.56 cm^2^, which dried in air for 35 min under ambient conditions. In this way, four sets of working electrodes for each type of concentration were prepared with a compact layer of Au-doped ZnO–Sm nanoparticles. Sintered the films at 400 °C for 35 min, and then the oven was shut off. When the temperature of the oven approached 80 °C, the working electrodes were taken out from the oven and soaked for sensitization in 0.5 mM solution of N719 dye in acetonitrile/tert-butanol (1:1, *v*/*v*) for 12 h. The sensitized photoelectrode films were removed from the dye solution and then air-dried at room temperature. Platinum-coated FTO glasses were used as counter electrodes. The cells were sealed by using laboratory parafilm, and the electrolyte (0.6 M 1-methyl propyl imidazolium iodide, 0.1 M lithium iodide, 0.05 M iodine and 0.5 M tert-butyl pyridine in acetonitrile) was injected through one of the holes made in counter electrode with a table drill machine. The hole then sealed with small squares of the microscopic slide to prevent the electrolyte leakage, as shown in Figure 2.

## 3. Results and Discussions

### 3.1. Structural Properties of Au-Doped ZnO–Sm Nanoparticles Films

**Figure 3:**
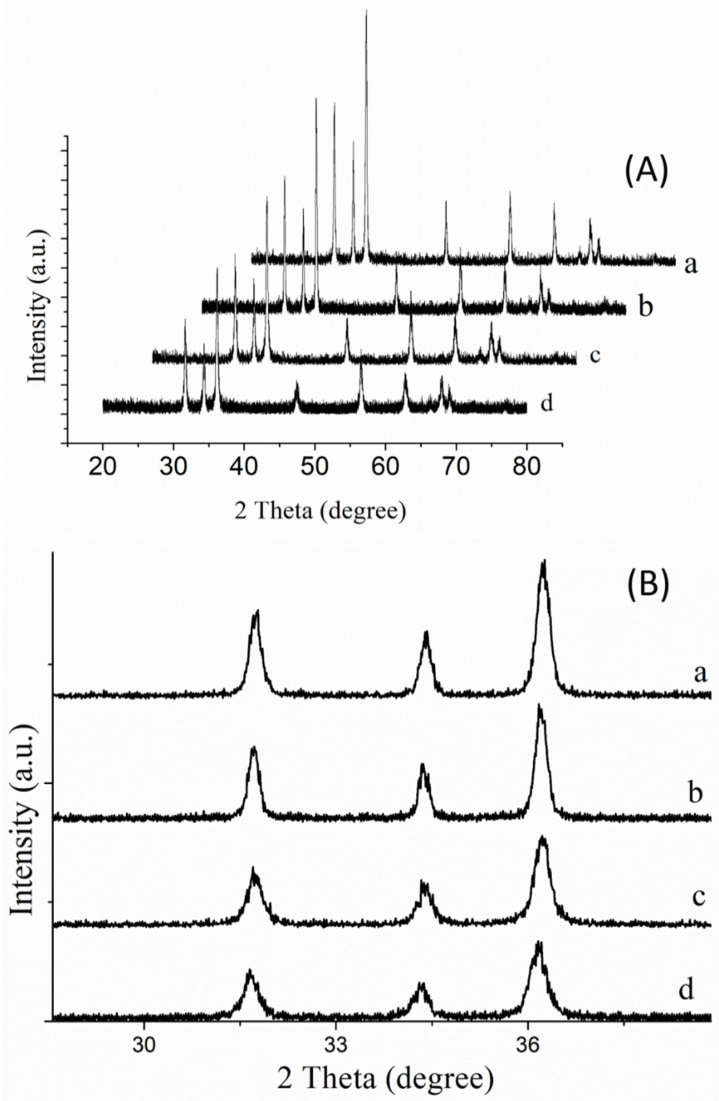
(**A**) XRD spectra of Au (0.0, 0.5, 1.0 and 1.5 wt %)-doped ZnO–Sm nanoparticle films. (**B**) Decrease in peak intensity.

**Figure 4:**
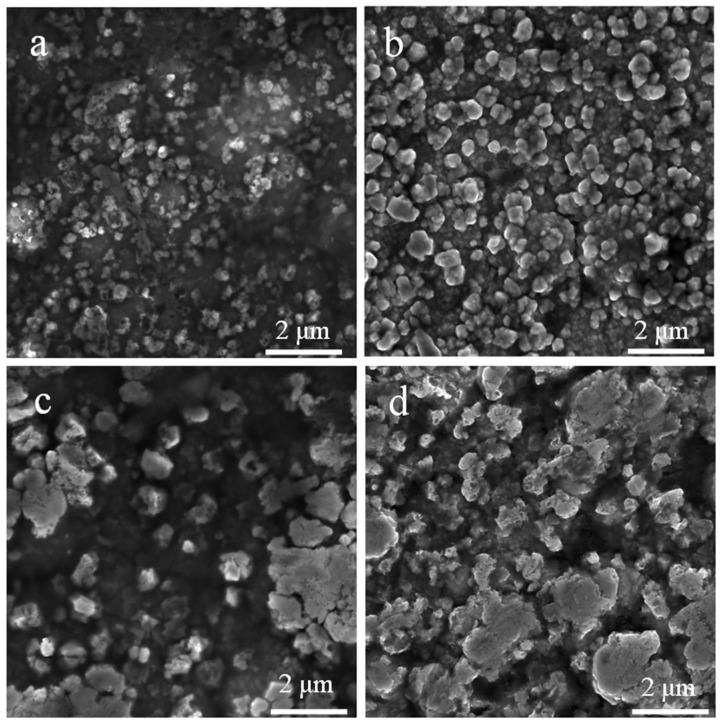
FESEM spectra of Au (0.0, 0.5, 1.0 and 1.5 wt %)-doped ZnO–Sm nanoparticle films.

XRD pattern of Au-doped ZnO–Sm nanoparticle films confirmed the wurtzite hexagonal single phase of ZnO with standard JCPDS card no-36–1451 in Figure 3A. As can be seen from Figure 3A (samples a–d), no extra diffraction peak as a result of doping of samarium and different concentration of gold up to 1.5 wt % was detected. The absence of characteristics peaks relating to Sm and Au in the XRD pattern may be due to the small quantity and appropriate incorporation of Sm^3+^/Au^+^ ions in the ZnO lattice [31]. This indicates the uniform substitution of Sm^3+^/Au^+^ ion either in the place of Zn^2+^ ion or on incorporation into the non-crystalline regions inside the lattice of ZnO. To explore the effect of doping materials on the crystallinity of ZnO film, the intensity of Bragg peaks (100), (002) and (101) were monitored. The intensity of peaks is decreased with the increased concentration of Au. Moreover, the intense Bragg Peaks slightly shift towards higher 2θ values, and their full width at half maximum (FWHM) was also increased for all Au-doped ZnO–Sm samples, as shown in Figure 3B. As the ionic radii of Au^+^ (1.37 Å) and Sm^3+^ (0.964 Å) are greater than Zn^2+^ (0.74 Å), therefore, peaks are shifted towards higher 2θ values. It means internal stresses are produced by this doping. Furthermore, this slight shift towards higher angle advocates that Sm^3+^/Au^+^ has been doped in ZnO in accordance with Vegard’s law [32]. In the previous literature: [33,34,35] have reported similar effects for Gd, Al and doped ZnO nanoparticles, respectively. However, [36,37] has observed that the Bragg peak is shifted towards a lower 2*θ* value by La-doping in ZnO. It also has been explained in the literature [38] that the deterioration in the crystalline quality of ZnO (as shown in Figure 4) may be due to the decreases in the intensity of Bragg peaks with the increase of Au concentration. This deterioration in crystal quality is attributed to the development of Zn-Au/Sm defects or the segregation of Sm/Au at the grain boundary. In [39] proposed that these changes in the crystallinity of ZnO may be due to the changes in the atomic environment with various concentrations of Au-doping or Sm doping. In [40] suggested that as ZnO has a closed packed hexagonal structure, there are empty octahedral sites, while Zn occupied half the tetrahedral sites. Therefore, sites are available in the ZnO structure in which both intrinsic Zn interstitials (Zni) and dopant atoms Sm/Au may occur. Moreover, the sharp diffraction peaks in the XRD pattern depict structural refinement and high crystallinity of the prepared samples.

### 3.2. Morphological Properties of Au-Doped ZnO–Sm Nanoparticles Films

**Figure 5:**
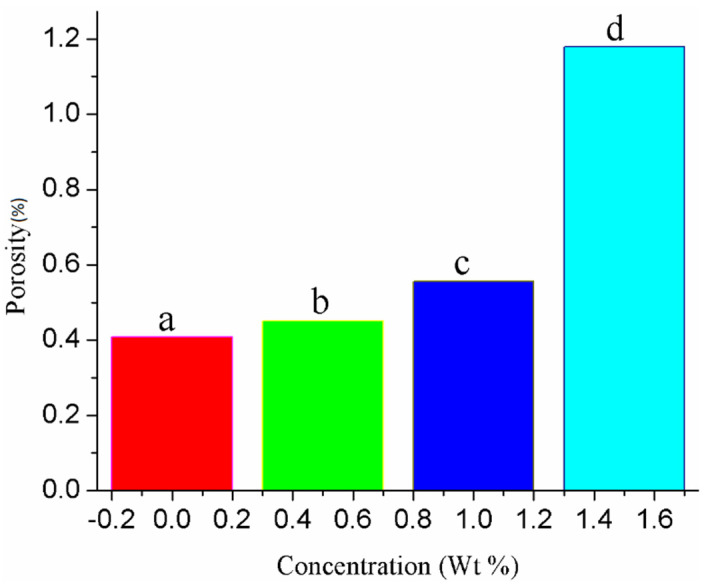
Porosity of Au (0.0, 0.5, 1.0 and 1.5 wt %)-doped ZnO–Sm nanoparticle films.

The surface morphologies of doped ZnO nanoparticle films were investigated by FE-SEM. In this research work, Au-doped ZnO–Sm nanoparticles have exhibited average particle size 0.11 μm for 0 wt % of Au, 0.15 μm for 0.5 wt % of Au, 0.99 μm for one wt % of Au and 1.81 μm for 1.5 wt % of Au. The average particle size of the Au-doped ZnO–Sm nanoparticles was estimated by Image J software((LOCI, University of Wisconsin, Madison, WI, USA). The variation in particle size of Au-doped ZnO–Sm nanoparticles can be explained by the agglomeration of nanoparticles. As the content of doping material increases, the morphology of the particles was changed from nanoparticles to aggregated particles. A similar trend in the agglomeration of Au-doped ZnO nanoparticles is obtained by [41]. In the present work, the obtained particle size is not uniform at all places of the samples. This is because the cluster of Sm and Au speckled from small to large value due to agglomeration, as shown in Figure 4. The cluster of Sm and Au particles are distributed on ZnO lattice, so the particle size is not uniform for host and dopant materials, and the product morphology is a mixture of small and aggregated particles. Due to the polydisperse size distribution of Au-doped ZnO–Sm aggregates, the porosity of the morphology increased, as is clear from Figure 5, which has been thought to be beneficial for more dye loading. This would result in an increase in the light-harvesting nature of the photoelectrodes as well as the power conversion efficiency of the DSSCs.

### 3.3. Optical Properties of Au-Doped ZnO–Sm Nanoparticle Films

**Figure 6:**
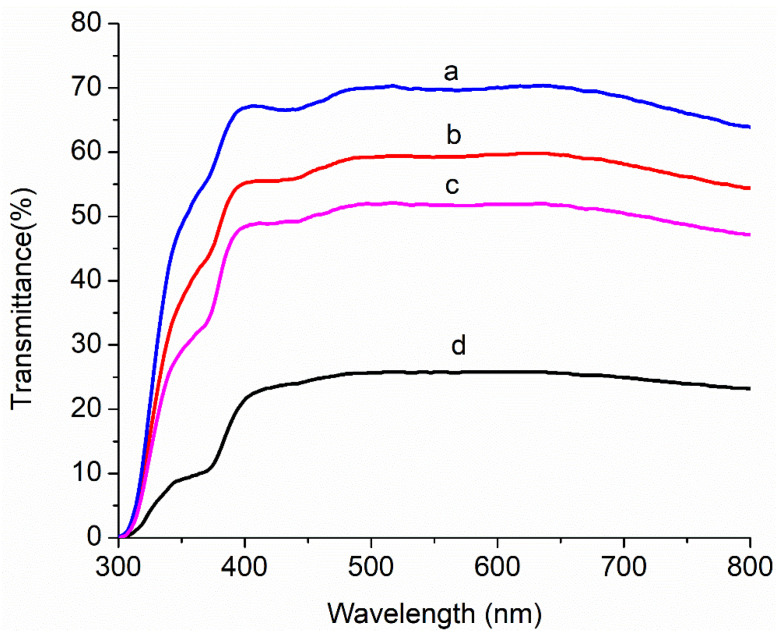
Transmittance spectra of Au (0.0, 0.5, 1.0 and 1.5 wt %)-doped ZnO–Sm nanoparticle films.

Figure 6 shows the optical transmittance with different concentrations (0.0, 0.5, 1.0 and 1.5 wt %) of Au-doped ZnO–Sm nanoparticle films. The optical transmittance was 25–70% in the wavelength range of 300 to 800 nm. With the increase in Au-doping from 0 to 1.5%, the transmittance continued to decrease to 25% for 1.5% Au-doped ZnO–Sm nanoparticle films. The decrease in transmittance was credited to the roughness and porous surface of doped films, as is clear from FESEM images in Figure 4. Sanjay et al. proposed that the decrease in transmittance may increase surface roughness, which was due to cluster agglomeration of Au/Sm with ZnO [42]. As the concentration of Au increasing in this study, more agglomeration occurs due to which larger particle size can be seen from Figure 6, which deteriorates the transparency and hence enhance the light absorption [43]. The interesting factor observed in this work is that the addition of a fixed ratio of Sm did not affect the transmittance. This behavior of Sm is good for solar cell efficiency with additional benefits to having more conductivity due to metallic character.

**Figure 7:**
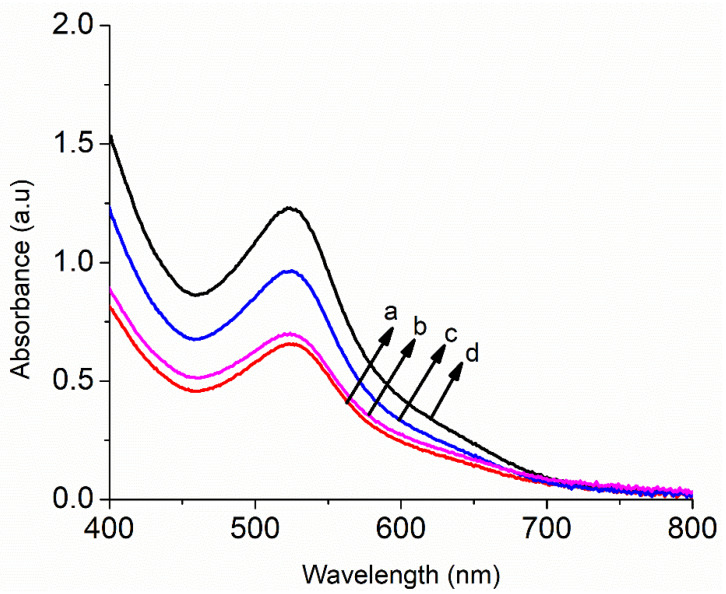
Absorbance spectra of Au (0.0, 0.5, 1.0 and 1.5 wt %)-doped ZnO–Sm nanoparticle films.

**Figure 8:**
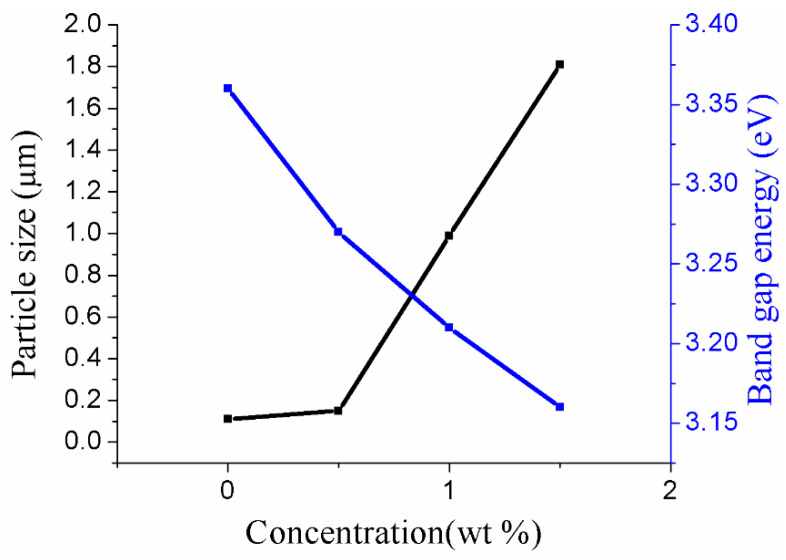
Particle size and bandgap energy of Au (0.0, 0.5, 1.0 and 1.5 wt %)-doped ZnO–Sm nanoparticle films.

Figure 7 demonstrates the optical absorbance spectra with different concentrations (0.0, 0.5, 1.0 and 1.5 wt %) of Au-doped ZnO–Sm nanoparticle films after N719 dye loading. It is seen from Figure 7 that the absorption was increased with doping of Sm and Au because of the doping morphology of small and large size nanoparticles in the visible light region. This increase in absorption may be due to the transfer of charge between the conduction or valence band of ZnO and the 4f level of Sm^3+^ ions. The peak of the sample with 0.5 wt% of Au-doped ZnO–Sm is found at 521 nm, which confirms dye loading. Although, for other concentrations of 1.0 wt % and 1.5 wt % of Au, the absorption peaks intensities of this peak are increased. This increase in absorbance exhibited a larger particle size. The increasing trend in absorption band from 521 nm to 530 nm confirmed that Au had modified the ZnO–Sm nanoparticles. The bandgap energies of the films were calculated before the N719 dye adsorption (not shown here) using the relation given [44]. The calculated values are given in Table 2. The narrowing of bandgap in semiconductors upon doping is a well-known general phenomenon. The energy levels created near the conduction band are due to donor impurities, and near the valence band is due to acceptor impurities. When the dopant amount has increased, then their density of states is increased and forms a continuum of states just like in the bands; as a result, Eg is decreased. Based on the literature, the Eg of the Au-doped ZnO–Sm is smaller than ZnO–Sm and slightly decreases with an increase in Au concentration. As Au is an IB-group element, its d orbital occupied energies are high near the O p level, while in the tetrahedral environment, both the orbits of Au d and O p have the symmetry of t_2_ [45,46]. When the Au atom occupies the Zn site, the strong d-p coupling between O and Au takes place, due to which the level of O 2p moves up and narrows the bandgap. Therefore, the Eg is decreased by Au doping. The particle size and Au concentration of different samples are shown in Figure 8.

### 3.4. I–V Measurements of Au-Doped ZnO–Sm Films

**Figure 9:**
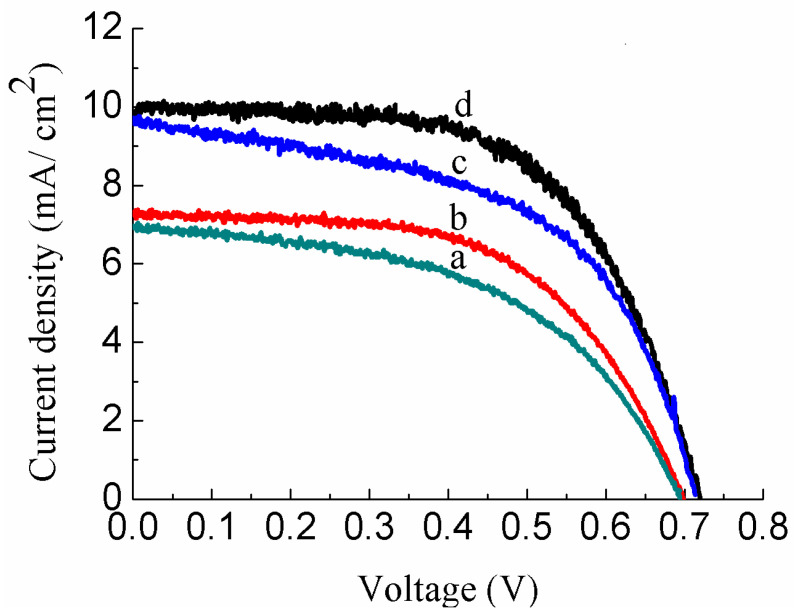
Photocurrent–voltage (J-V) characteristics of Au (0.0, 0.5, 1.0 and 1.5 wt %)-doped ZnO–Sm nanoparticle films.

I–V measurements of the DSSCs fabricated with different concentrations of Au-doped ZnO–Sm nanoparticle films are shown in Figure 9. The short-circuit current density (J_SC_), open-circuit voltage (V_OC_), fill factor (FF), and power conversion efficiency (*η*) of these cells are given in Table 3. From the parameters, the cell fabricated with 0.5 wt % of Au-doped ZnO–Sm has an obvious enhancement of photocurrent and efficiency (i.e., J_SC_ = 7.02, *η* = 3.26) compared with the cell fabricated with 0.0 wt % of Au-doped ZnO–Sm (i.e., J_SC_ = 6.96, *η* = 2.47%) due to the presence of Au, which acts as blocking layer to decrease the recombination rate and increase the transport of electrons. There is an increase of 76 % in efficiency. Furthermore, the DSSCs fabricated with 1.0 wt % and 1.5 wt % of Au-doped ZnO–Sm have an optimal performance (i.e., increase in J_SC_ = 38% and 43%, and in *η* = 50% and 76%, respectively) as indicated in Table 3. This improvement in the DSSCs parameters can be ascribed in two ways. One way is the up/down-conversion process in which UV and NIR radiations can be shifted to visible light due to the doping of RE ions in ZnO. It is known that the RE-doped ZnO absorbs two or more low-energy photons and then emits high-energy photons locating in the main absorption region of the dye N719 [25] because the dye N719 have strong absorption around 550 nm [24]. This widening of the absorption region of the cell is the main reason for enhancing the light to the power conversion efficiency of DSSCs. In addition, RE doping also acts as a blocking layer that inhibits the charge recombination between photoelectrode and $I^{-}/I_{3}^{-}$, which suppressed the dark current efficiently for DSSCs. A second way to boost the performance of the 0.5 wt %, 1.0 wt % and 1.5 wt % of Au-doped ZnO–Sm-based DSSCs is attributed to the presence of the ZnO–Sm/Au-blocking layer, which blocks the back electron transfer from the conduction band of ZnO to $I^{-}/I_{3}^{-}$, redox electrolyte [26]. Therefore, the electrons flow towards the oxidized dye molecules or the redox electrolyte, thereby leading to an improvement in the DSSCs parameters [24]. According to [27], blocking layers exist in the ZnO–Sm/Au interface because of the greater work function of Au (5.1 eV) than the electron affinity of ZnO (4.2 eV), which prevents the backward transfer of electrons and pushes the electron towards either the excited dye molecules or the liquid electrolyte. As a consequence, the photocurrent of the DSSCs might be increased efficiently. In addition, it was observed that the doping of RE ions and different concentration of Au have a great impact on the performance of DSSCs by controlling the backward transfer of electrons and making useful UV and NIR spectrum of light by up/down-conversion process.

### 3.5. Impedance Spectroscopy Measurements of Au-Doped ZnO–Sm Films

**Figure 10:**
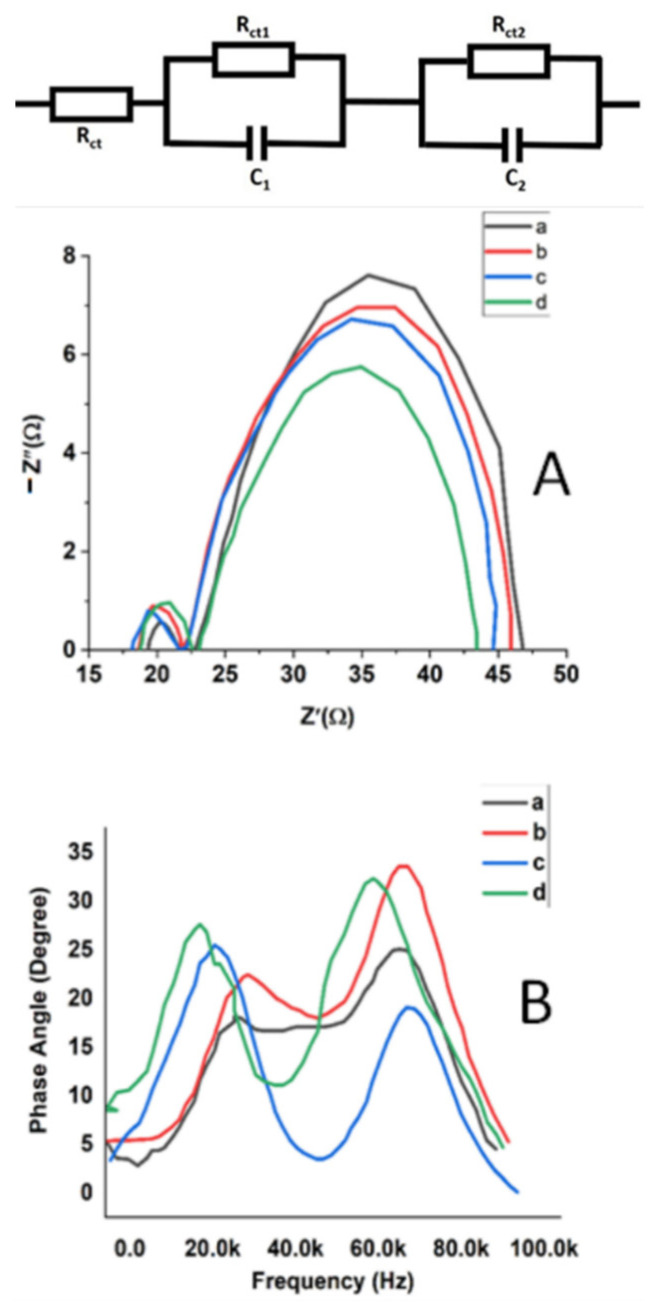
(**A**) Nyquist plots and (**B**) Bode plot of Au (0.0, 0.5, 1.0 and 1.5 wt %)-doped ZnO–Sm nanoparticle films.

To elucidate the dissimilarities in the charge transport phenomena of Au-doped ZnO–Sm film-based DSSCs with different concentrations of Au (0.0, 0.5, 1.0 and 1.5 wt %), electrochemical impedance spectroscopy (EIS) was utilized. Generally, Nyquist plots of DSSCs show three semicircles. However, in the present work, two semicircles were observed shown in Figure 10 A. These two semicircles in the Nyquist plot were attributed to the low resistance of ion transport in the redox-electrolyte. An equivalent circuit model was applied to analyze the charge-transport process in Au-doped ZnO–Sm photoanodes, and the resulting impedance parameters are listed in Table 3. Wang et al. [47] suggested that the first semicircle in high-frequency range was ascribed to charge transfer resistance (R_ct1_) and chemical capacitance (C_1_) at the redox electrolyte/Pt-coated counter electrode interface. While the second semicircle in the low-frequency range was due to electron transfer, transport resistance (R_ct2_) and chemical capacitance (C_2_) at the Au-doped ZnO–Sm/dye/electrolyte interface. However, Rs stands for the sheet resistance at the FTO/ZnO–Sm interface. DSSCs based on Au-doped ZnO–Sm photoanodes with different concentrations of Au (0.0, 0.5, 1.0 and 1.5 wt %) display similar values of R_ct1_ because of the use of the same counter electrode (Pt/FTO glass) and electrolyte. On the other hand, R_ct2_ gradually decreases with the increase of concentration and has the lowest value for the DSSC doped with 1.5 wt % of Au. The decrease in R_ct2_ with an increase of Au concentration is due to the injection of more electrons in the ZnO conduction band because of making blocking layer. This blocking layer inhibits the backward transfer of an electron from the conduction band of ZnO to redox-electrolyte, resulting in the enhanced light-harvesting capability. This enhancement of the photocurrent for all Au-doped ZnO–Sm phot anodes is in good agreement with I–V data.

The Bode phase plot of electrochemical impedance spectroscopy for Au-doped ZnO–Sm nanoparticles photoanodes with different concentrations of Au (0.0, 0.5, 1.0 and 1.5 wt %) is presented in Figure 10B. These spectra show the frequency (*f*) peaks of the charge-transport process at different interfaces for all the DSSCs. It is clear from Figure 10B that the frequency (*f*) in the region between 0.0 and 40.0 kHz is associated with the electron lifetime (*τ*) through *τ* = 1/*ω* = 1/2π*f* [48,49,50]. In this equation, f and *τ* are related to the charge transfer at Au-doped ZnO–Sm dye/electrolyte interface, and the calculated results for the electron lifetimes are listed in Table 3. It can be inferred (clearly seen) from the plot that with the increase of Au concentration, the peak positions for the photoelectrodes composed of Au-dope ZnO–Sm nanoparticles are shifted to the low-frequency region. With the increase of Au concentration, an increased trend of electrons lifetime (τ) was observed due to the decrease in electron trap states in the conduction band of Au-doped ZnO–Sm nanoparticles photoanodes. DSSC fabricated with 1.5 wt % of Au-doped ZnO–Sm has low resistance R_ct2_ and long electrons lifetime (*τ*), could support the transport of electron through long-distance and inhibit the backward transfer of electrons from CB of ZnO to redox electrolyte. Therefore, from our results, we can conclude that the DSSC fabricated with Au-doped ZnO–Sm photoanodes showed improved photo-induced current. The improvement was gained through the up/down-conversion of RE ions and Au doping, which enhanced charge-transport properties and prohibited the backward flow of electrons.

## 4. Conclusions

Au-doped ZnO–Sm nanoparticle films were successfully synthesized with different concentrations of Au (0.0 wt % to 1.5 wt %) and fixed concentrations of Sm (1 wt %). XRD spectra showed that prepared samples were pure and crystalline, and no impurity peaks were observed in the samples; however, there is little shift in the peak position, i.e., internal stresses are produced by this doping. SEM morphologies showed that due to agglomeration, the particle size increases with the increase of Au concentration. Due to the increase in particle size, porosity also increases, which in turn increases dye loading and light-harvesting. Transmission spectra showed a decreasing trend because of the roughness and porous surface, whereas the absorbance increases due to small and large size nanoparticles in the visible light region. These films were used as photoanodes to boost the efficiency of DSSCs by controlling the backward transfer of electrons (enhance electron transport) and increase the absorption spectrum of light by the up/down-conversion process. The cell fabricated with 1.5 wt % of Au-doped ZnO–Sm photoanode showed the highest efficiency of 4.35%, which is about 76% higher compared with their other counterparts. EIS measurements infer that the improvement in the cell performance could be due to the enhanced light spectrum and faster electron transport.

## Figures and Tables

**Table 1:** Parameters specifying fabrication of Au (0.0, 0.5, 1.0 and 1.5 wt *%*)-doped ZnO–Sm nanoparticles.

Samples	Content of ZnO (wt *%*)	Content of Sm (wt *%*)	Content of Au (wt *%*)
a	100	1	0.0
b	100	1	0.5
c	100	1	1.0
d	100	1	1.5

**Table 2:** Grain size and bandgap energy of Au (0.0, 0.5, 1.0 and 1.5 wt %)-doped ZnO–Sm nanoparticle films.

Samples	Content of ZnO (wt %)	Content of Sm (wt %)	Content of Au (wt %)	Particle Size (μm)	Band Gap Eg (eV)
a	100	1	0.0	0.11	3.36
b	100	1	0.5	0.15	3.27
c	100	1	1.0	0.99	3.21
d	100	1	1.5	1.81	3.16

**Table 3:** I–V and electrochemical impedance spectroscopy (EIS) measurements of Au (0.0, 0.5, 1.0 and 1.5 wt %)-doped ZnO–Sm nanoparticles Based DSSCs.

Cell Parameters	Samples			
	a	b	c	d
J_SC_ (mA/cm^2^)	6.96 ± 0.018	7.02 ± 0.056	9.63 ± 0.022	9.97 ± 0.019
V_OC_ (V)	0.694 ± 0.003	0.699 ± 0.004	0.714 ± 0.002	0.719 ± 0.004
FF	0.52 ± 0.003	0.59 ± 0.005	0.54 ± 0.004	0.61 ± 0.003
*η* (%)	2.47 ± 0.002	3.26 ± 0.013	3.70 ± 0.003	4.35 ± 0.002
R_ct2 (Ω)_	130.62 ± 0.14	113.84 ± 0.10	96.47 ± 0.21	90.57 ± 0.16
*τ* (ms)	16 ± 1	21 ± 3	32 ± 1	39 ± 2

## Data Availability

The raw data used for this proposed work were cited in the manuscript. Moreover, the derived data supporting the findings of this study were graphically depicted and are available with the corresponding author on request.
